# Insights from quantitative and mathematical modelling on the proposed 2030 goals for Yaws

**DOI:** 10.12688/gatesopenres.13078.1

**Published:** 2019-10-16

**Authors:** Louise Dyson, Eric Q. Mooring, Alex Holmes, Michael J. Tildesley, Michael Marks

**Affiliations:** 1 Mathematics Institute, University of Warwick, Coventry, CV4 7AL, UK; 2 School of Life Sciences, University of Warwick, Coventry, CV4 7AL, UK; 3 Department of Epidemiology, Harvard T.H. Chan School of Public Health, Boston, MA, USA; 4 Clinical Research Department, Faculty of Infectious & Tropical Diseases, London School of Hygiene & Tropical Medicine, London, UK

**Keywords:** yaws, modelling, neglected tropical diseases

## Abstract

The World Health Organization is currently developing 2030 goals for neglected tropical diseases (NTDs). In these, yaws has been targeted for eradication by 2030, with 50% of member states certified free of yaws transmission by 2023. Here we summarise the yaws modelling literature and discuss the proposed goal and strategy. The current Morges strategy involves rounds of Total Community Treatment (TCT), in which all members of the community are treated, and Total Targeted Treatment (TTT), treating active cases and their contacts. However, modelling and empirical work suggest that latent infections are often not found in the same household as active cases, reducing the utility of household-based contact tracing for a TTT strategy. Economic modelling has also discovered uncertainty in the cost of eradication, requiring further data to give greater information. We also note the need for improved active surveillance in previously endemic countries, in order to plan future intervention efforts and ensure global eradication.

## Disclaimer

The views expressed in this article are those of the author(s). Publication in Gates Open Research does not imply endorsement by the Gates Foundation.

## Background

Yaws is a bacterial infection causing lesions in the skin and bones, primarily found in tropical areas. It is caused by the bacteria *Treponema pallidum pertenue*, and spread by skin to skin contact with an infected lesion. While the initial lesion (“mother yaw”) may spontaneously heal without treatment, the individual remains infected and relapses to infectious yaws represent a reservoir of future infection. Such relapses can occur up to 10 years later, and symptoms deteriorate with additional relapses. Without treatment, progression to secondary and tertiary yaws can result in chronic disfigurement and disability. It is critical, therefore that any eradication strategy must successfully treat asymptomatic as well as infectious individuals.

Efforts to eradicate yaws in the 1950s used injections of benzylpenicillin to successfully reduce worldwide cases by 95% ( Mitjà *et al.*, 2015). However, limited surveillance and resources unfortunately lead to resurgence in West Africa, Asia and the Pacific in the 1970s ( Asiedu *et al.*, 2014). Renewed eradication efforts began in 2012, when a single dose of oral azithromycin was shown to be non-inferior to injection with benzylpenicillin ( Mitjà *et al.*, 2012). In 2016 India became the first country to be declared by the World Health Organization (WHO) to be free of yaws.

Current eradication efforts follow the Morges strategy ( World Health Organization, 2012), which recommends one or more rounds of total coammunity treatment (TCT) followed by rounds of total targeted treatment (TTT), in which active cases and their contacts (household, frequent family friends, schoolmates and playmates) are treated. TTT is intended to ensure treatment of asymptomatic non-infectious individuals (latent infections), who can later relapse to infectious yaws. Azithromycin is effective in over 95% of cases, with intramuscular benzathine penicillin used as a second line treatment where necessary.

The WHO is currently developing the 2030 neglected tropical diseases (NTDs) Roadmap, detailing the goals for each disease and the strategies that should be used to reach these goals. Two rounds of consultations with endemic countries, partners, donors and other stakeholders were held to refine the proposed targets. The goal for yaws is to certify all member states free of yaws transmission by 2030, with 50% certified by 2023 and 70% by 2025 ( World Health Organization, 2019). In this letter we discuss the insights gained from modelling work to date, the feasibility of the proposed goals and the strategies required to reach them.

## Mathematical modelling of yaws transmission

The mathematical modelling of yaws transmission is still in its early days, comprising just seven papers that include modelling ( Dyson *et al.*, 2018; Fitzpatrick *et al.*, 2014; Fitzpatrick *et al.*, 2018; Gart & De Vries, 1966; Marks *et al.*, 2017; Mooring *et al.*, 2019; Mushayabasa *et al.*, 2012) and a single example fitting a simple catalytic model to age structured yaws data ( Muench, 1959). While some work comprises mostly theoretical research ( Gart & De Vries, 1966; Mushayabasa *et al.*, 2012), more recent publications have used economic or transmission modelling to consider questions regarding the funding and implementation of eradication programmes.

## The costs of yaws eradication

Economic modelling undertaken by Fitzpatrick *et al.* (2014) revealed a high degree of uncertainty in the cost of eradication, costing USD 362 (75-1073) million in 12 endemic countries and USD 324 (47-522) million per disability-adjusted life year. This is calculated using a compartment model with primary, secondary and tertiary infection with gradual exit of the at risk population due to poverty reduction. There are many uncertain quantities in this calculation due to lack of data regarding the cost of TTT (here assumed to be 30-50% of the cost of TCT), lack of disability weights for early or late-stage yaws (DALYs were calculated using weights for comparable conditions) and uncertainties around the population at risk and the unit costs of delivery in different countries.

## Campaign strategies: TCT vs TTT

Two recent papers focussed on the feasibility of reaching eradication by the current strategy, and ways to determine how many rounds of TCT and TTT will be required. The first uses a stochastic model of community-level yaws transmission, and determined that to reach 80% probability of achieving eradication using 8 rounds of twice-yearly treatment (3 rounds of TCT followed by 5 rounds of TTT) 80% coverage in low transmission (R0 = 1.45) settings, and 95% coverage in high transmission (R0 = 2.47) settings was required ( Marks *et al.*, 2017). Here TTT is modelled as having the potential to achieve different coverage levels in active and latent yaws. If only one round of TCT was conducted then 90% coverage was required, followed by 5 rounds of TTT at 90% coverage of active infections and 65% coverage of latent infections. In high transmission settings with yearly treatment no combination of variables or treatments achieved elimination of transmission (EOT) in the Marks *et al.* model.

A second paper used household model of infection, parameterised using data from the Solomon Islands, and indicated that latent infections are often not co-located in the same households as clinical infections. This would imply that treating clinical cases and their household contacts would miss a large fraction of latent infections ( Dyson *et al.*, 2018). This work inferred the infection statuses of adults, for whom serological tests were not conducted and found that between 65% and 100% of latent infections were in households without a clinical infection present. The conclusion that latent infections are not closely connected to active cases is also supported by empirical studies showing that there may be as many as 6–10 latent cases for each active yaws case ( Marks *et al.*, 2015) and that in an implementation study of the Morges strategy ( Mitjà *et al.*, 2015) the ratio of latent:active cases increased following several rounds of treatment suggesting that the intervention (single round of TCT followed by only TTT) was more effective at treating active cases than latent cases. Since latently infected individuals may relapse to infectious yaws as many as 10 years later, this represents a significant reservoir of infection. Indeed, even the time to relapse is uncertain, so that it is unclear how long it is necessary to observe zero cases for before we can be certain of elimination. Note that in the household model, even with 100% coverage of active cases, household-based TTT would achieve a maximum coverage in latent infections of 35% ( Dyson *et al.*, 2018), far below the 65% coverage of latent infection required in the Marks *et al.* model.

A recent stochastic compartmental metapopulation model constructed by Mooring *et al.* (2019) also examined the performance of TCT vs TTT in reducing the prevalence of both active and latent yaws. The model simulated yaws transmission and intervention in populations averaging 20000 people divided among 200 “hamlets” that ranged in size from 75 to 125 people. In view of the finding from other modelling studies that a household definition of contact may be inadequate, the model of TTT assumed that cases of active disease were detected with a probability of 90% and that treatment was administered to, on average, 90% of all persons living in any hamlet with at least one detected active case. The model assessed the number of rounds of TTT required to achieve the same impact as an additional round of TCT across varying assumptions about spatial heterogeneity in transmissibility, mixing between hamlets, and the initial number of rounds of TCT (1 to 3). When the overall prevalence of yaws (both active and latent infection combined) was the outcome of interest, this model demonstrated that multiple rounds of TTT were likely to be required to match the impact of a single additional round of TCT even when TTT was administered to the entire population of a village.

## Yaws surveillance

The currently proposed goal for yaws is eradication by 2030, defined as interruption of transmission (absence of new cases) globally. This raises questions regarding the proposed strategy and means of certifying elimination. Recent regression modelling estimates that, for 66 out of the 86 countries whose current status is unknown, there is a less than 50% probability of reporting cases without an active surveillance program ( Fitzpatrick *et al.*, 2018). Thus this indicates that to ensure global eradication by 2030, it is necessary to undertake surveillance in these previously endemic countries. Furthermore, surveillance during elimination campaigns is hindered by the cross-reactivity of the yaws serological test, so that only children can be reliably classed as having latent yaws from the testing. We note, however, that it is still the case that adults that are serologically negative are not carriers of latent yaws, and therefore tests in adults may still be of use.

## Where are there risks that need to be mitigated to achieve the goal?

The primary risk for yaws, is a general lack of political commitment and funding for yaws eradication. This lack of community buy-in and serious questions regarding whether yaws eradication should be a priority presents a serious risk to program success. The possibility of achieving eradication by 2030 is also highly dependent on what proportion of previously-endemic countries still contain cases of yaws as this will have significant implications for the scale of intervention required globally. In recent years two countries (Liberia and the Philippines) have been found to still be endemic ( World Health Organization, 2018) highlighting the possibility that the number of countries requiring interventions for yaws eradication may grow substantially as surveillance is undertaken in a wider range of settings.

Systematic non-participation in both TCT and TTT may pose a hindrance to elimination. No measure has been made in this disease; however, previous modelling work shows that in mass drug administration campaigns systematic non-participation can have a significant effect, particularly when levels of reinfection are low ( Dyson *et al.*, 2017). In the wider literature on MDA in infectious diseases, correlations between rounds were found of between 0.28 and 0.54 ( Dyson *et al.*, 2017). Future modelling work could determine the effect of systematic non-participation on the outcome of elimination campaigns for yaws. In addition, drug resistance represents a risk to implementation of TCT and TTT with azithromycin. Resistance has been reported in Papua New Guinea in the context of a yaws eradication study ( Mitjà *et al*., 2018). Finally, as with many NTDs, it is important to require sufficient coverage of surveillance efforts to be sure that zero cases found represents interruption of transmission. As discussed above, in many countries it is unlikely that passive surveillance will result in reporting of new cases ( Fitzpatrick *et al.*, 2018).

## Future work

Current modelling work is limited by the availability of suitable datasets, since a model fitted to data from a specific geographical location may not be directly applicable in another setting. In particular high-quality survey data is lacking from West and Central Africa, the region of the world where the second most cases arise (after the Pacific, where most current modelling data is derived from). It is this lack of setting-specific data that hinders country-specific predictions. In addition, given suitable data, modelling could also be undertaken that includes definitions of “contact” for TTT other than household contacts. For example, modelling school based contacts requires data in which the schools attended by children is included.

Other potential extensions include age-prevalence modelling, to determine whether serological testing in children aged 1–5 is necessary and sufficient for determining the interruption of transmission. Along with spatial modelling to assess clustering, these models can be used to consider surveillance strategies. So far, most yaws modelling has focused on TCT and TTT, and not ongoing surveillance. Additional research should consider in greater depth ongoing community-based surveillance and the implications of treating suspected cases and, possibly, their contacts. Such research would shed light on the extent to which rapid identification of symptomatic cases should be a programmatic priority.

The dynamics of latent infection (i.e. the period during which infected individuals are at risk of reactivating and developing symptomatic and infectious yaws) deserves further study. Future modeling studies should further explore the extent to which results are sensitive to assumptions about the duration of latent infections. Most yaws models have implicitly assumed that the duration of latent infection is exponentially distributed. This distributional assumption deserves further study. Measuring the duration and distribution of latent infection is challenging, in part because it would be unethical to prospectively follow-up untreated yaws patients. As a first step, models could be used to explore what sort of data would be required to indirectly infer the dynamics of latent infection.

Finally, further modelling may be undertaken to answer the question: “would achieving >90% coverage in children even if the overall population coverage is <90% be sufficient to interrupt transmission?” This work would need additional data regarding the amount of transmission between children and adults, which is difficult to obtain due to the problems with serological testing in adults. Without such data we could still ask the question: if adult transmission is very frequent we wouldn’t expect focusing coverage on children to interrupt transmission - how much rarer does it need to be for this strategy to be successful? Further economic modelling could also be undertaken given more data regarding the costs of TTT.

## Data availability

No data are associated with this article.
