# Supplementary material for: Insights from quantitative and mathematical modelling on the proposed 2030 goals for Yaws
Source: Gates Open Res. 2019 Oct 16;3:1576. [Version 1] doi: 10.12688/gatesopenres.13078.1 (PMC6913063; doi:10.12688/gatesopenres.13078.1)
Supplement: Peer review history

# Peer review: Reviewer response for version 1

**DOI:** 10.21956/gatesopenres.14217.r28252

**Published:** 2019-12-12

**Authors:** David Šmajs, Petra Pospíšilová

**Affiliations:** 1 Department of Biology, Faculty of Medicine, Masaryk University, Brno, Czech Republic

The article summarizes 7 studies published on modelling of transmission of yaws, a disease targeted for eradication by WHO by 2030. Recently, some countries were declared to be free of yaws (India and Ecuador) indicating that elimination of yaws is an attainable goal. However, many factors affecting an achievement of this goal are not completely understood. The authors are well aware of the lack of the data allowing to specifically model yaws transmission in different geographical locations and to propose optimal eradication strategies for different countries or regions. The paper highlights main risks that can contribute to failure of the eradication efforts including lack of political commitment and funding, systematic non-participation in both TCT and TTT, selection of suboptimal strategy based on single or insufficient number of TCT rounds followed by TTT, possible emergence of azithromycin resistance of the yaws agent and the lack of sufficient surveillance to prove interruption of transmission. However, there are other potential risks that need to be considered and these include: 1) the role of migration and travel in reintroduction of infection to the treated population, 2) limited knowledge on proportion of latent and active yaws cases, efficiency of the treatment of latent yaws and its transmission routes in many areas and 3) the potential risk of transmission of yaws agent from non-human primates that were shown to have treponemal infections caused by strains similar to those causing yaws.

 There are many studies describing a short-term effect of azithromycin MDA on reduction of active and latent yaws cases (e.g., Ghinai *et al.*, 2015 ^1^, Marks *et al.*, 2015 ^2^), however, there is only one longitudinal study using molecular detection and typing of *Treponema pallidum* subspecies *pertenue* (Mitja *et al.*, 2018 ^3^). This study showed that the active cases detected 42 months post single MDA round include the following patient categories: travelling residents, newly arrived migrants, MDA-treated non-travelling residents, and non-travelling residents absent from initial MDA. Most of the cases identified at this point were genotyped and belonged to the most frequent genotype found throughout the whole study. Although failure to achieve elimination of yaws is thought to be caused mainly by the proportion of individuals absent at the time of mass treatment, the presented facts indicate that other factors can contribute to this failure (in-migration, reinfection from other active cases, failure of treatment of latent cases).

 As shown in the study of Mitja *et al.* (2017 ^4^), the serological cure of yaws was achieved in the same proportion of active and latent cases after 24 months. The data presented by Mitja *et al.* (2018 ^3^) showed that children with high titer latent yaws aged 6-15 years lowered from 19% at baseline to 7% at 42 months. However, it is not known if all the children were treated or not. Studies concentrating on the follow-up of latent cases should help to elucidate the treatment efficiency and dynamics of latent yaws infections as suggested by the authors of the open letter. The proportion of latent cases in the one round of TCT followed by 7 TTT treated population remains very high (> 5%) and represents potential risk of re-emergence of yaws. The successful strategy for elimination of yaws will have to implement additional steps to effectively target the remaining latent cases and the potential spread of yaws.

 In recent years, reports describing infections of non-human primates with treponemes highly similar to the agent of human yaws have been published (Knauf *et al.*, 2018 ^5^, Chuma *et al.*, 2019 ^6^). Although the transfer from animals to humans has not been described yet, an active surveillance in areas of the risk should be planned as a part of the global yaws eradication program.

 The planning of the yaws eradication program therefore requires future studies to predict the optimal ratio between TCT and TTT rounds, extent of TTT treatment, prediction of eradication costs and prediction of optimal strategy of active surveillance in the involved states and areas.

We confirm that we have read this submission and believe that we have an appropriate level of expertise to confirm that it is of an acceptable scientific standard.

## References

[1] : A cross-sectional study of 'yaws' in districts of Ghana which have previously undertaken azithromycin mass drug administration for trachoma control. *PLoS Negl Trop Dis*.2015;9(1) : 10.1371/journal.pntd.0003496 e0003496 10.1371/journal.pntd.0003496 25632942PMC4310597

[2] : Impact of Community Mass Treatment with Azithromycin for Trachoma Elimination on the Prevalence of Yaws. *PLoS Negl Trop Dis*.2015;9(8) : 10.1371/journal.pntd.0003988 e0003988 10.1371/journal.pntd.0003988 26241484PMC4524711

[3] : Re-emergence of yaws after single mass azithromycin treatment followed by targeted treatment: a longitudinal study. *The Lancet*.2018;391(10130) : 10.1016/S0140-6736(18)30204-6 1599-1607 10.1016/S0140-6736(18)30204-6 PMC592072229428183

[4] : Effectiveness of single-dose azithromycin to treat latent yaws: a longitudinal comparative cohort study. *The Lancet Global Health*.2017;5(12) : 10.1016/S2214-109X(17)30388-1 e1268-e1274 10.1016/S2214-109X(17)30388-1 29107621

[5] : Nonhuman primates across sub-Saharan Africa are infected with the yaws bacteriumTreponema pallidum subsp.pertenue. *Emerging Microbes & Infections*.2018;7(1) : 10.1038/s41426-018-0156-4 1-4 10.1038/s41426-018-0156-4 30228266PMC6143531

[6] : Strain diversity of Treponema pallidum subsp. pertenue suggests rare interspecies transmission in African nonhuman primates. *Sci Rep*.2019;9(1) : 10.1038/s41598-019-50779-9 14243 10.1038/s41598-019-50779-9 31578447PMC6775232

# Peer review: Reviewer response for version 1

**DOI:** 10.21956/gatesopenres.14217.r28141

**Published:** 2019-11-27

**Authors:** Caroline Trotter

**Affiliations:** 1 Disease Dynamics Unit, Department of Veterinary Medicine, University of Cambridge, Cambridge, UK

Overall I found this article to be clearly structured and well written. There is not a large body of work on modelling yaws and the authors highlight that there is much more to be done. A key problem is the lack of data availability from certain regions to which models can be fitted. Given the uncertainties in the current costs of eradication, I wonder if the authors would consider proposing a value of information study to help target new data collection?  

 I noted one error - in the section "The costs of yaws eradication",  the authors quote USD 324 million per DALY, whereas in fact the source cites USD 324 per DALY (a much more reasonable figure).

I confirm that I have read this submission and believe that I have an appropriate level of expertise to confirm that it is of an acceptable scientific standard.
